# Characterization of the Complete Mitochondrion Genome of Diurnal Moth *Amata emma* (Butler) (Lepidoptera: Erebidae) and Its Phylogenetic Implications

**DOI:** 10.1371/journal.pone.0072410

**Published:** 2013-09-12

**Authors:** Hui-Fen Lu, Tian-Juan Su, A-Rong Luo, Chao-Dong Zhu, Chun-Sheng Wu

**Affiliations:** 1 Key Laboratory of Zoological Systematics and Evolution, Institute of Zoology, Chinese Academy of Sciences, Beijing, China; 2 University of Chinese Academy of Sciences, Beijing, China; University of South Florida College of Medicine, United States of America

## Abstract

Mitogenomes can provide information for phylogenetic analyses and evolutionary biology. The complete mitochondrial genome of *Amata emma* (Lepidoptera: Erebidae) was sequenced and analyzed in the study. The circular genome is 15,463 bp in size, with the gene content, orientation and order identical to other ditrysian insects. The genome composition of the major strand shows highly A+T biased and exhibits negative AT-skew and GC-skew. The initial codons are the canonical putative start codons ATN with the exception of *cox1* gene which uses CGA instead. Ten genes share complete termination codons TAA, and three genes use incomplete stop codons TA or T. Additionally, the codon distribution and Relative Synonymous Codon Usage of the 13 PCGs in the *A. emma* mitogenome are consistent with those in other Noctuid mitogenomes. All tRNA genes have typical cloverleaf secondary structures, except for the *trnS1* (AGN) gene, in which the dihydrouridine (DHU) arm is simplified down to a loop. The secondary structures of two rRNA genes broadly conform with the models proposed for these genes of other Lepidopteran insects. Except for the A+T-rich region, there are three major intergenic spacers, spanning at least 10 bp and five overlapping regions. There are obvious differences in the A+T-rich region between *A. emma* and other Lepidopteran insects reported previously except that the A+T-rich region contains an ‘ATAGA’ -like motif followed by a 19 bp poly-T stretch and a (AT)_9_ element preceded by the ‘ATTTA’ motif. It neither has a poly-A (in the α strand) upstream *trnM* nor potential stem-loop structures and just has some simple structures like (AT)_n_GTAT. The phylogenetic relationships based on nucleotide sequences of 13 PCGs using Bayesian inference and maximum likelihood methods provided a well-supported a broader outline of Lepidoptera and which agree with the traditional morphological classification and recently working, but with a much higher support.

## Introduction

The ancestral insect mitogenome is a closed-circular DNA molecule, spanning 16–20 kilobases (kb) [1], containing 13 protein-coding genes (PCGs), two ribosomal RNA genes (rRNAs), and 22 transfer RNA genes (tRNAs). It also has a control region (A+T-rich region) of highly variable length, which regulates the transcription and replication of the genome [2]. Twenty three genes are coded on the majority strand while the rest are coded on the minority strand. Because of the characteristics of small size, maternal inheritance, relatively rapid evolutionary rate, lack of introns and genetic recombination, the mitochondrial DNA (mtDNA) has been widely used in studies on molecular evolution, molecular phylogenetics and population genetics [3]–[5]. Mitochondrial genomes (mtgenomes) are very important subject for different scientific disciplines including animal health, comparative and evolutionary genomics, molecular evolution, phylogenetic and population genetics [3].

Lepidoptera (moths and butterflies) is the second largest order in Insecta,containing over 155 000 described species [6], [7]. In Lepidoptera, Noctuoidea is the largest superfamily with about 42,400 species worldwide [7], [8]. Despite such huge taxonomic diversity the existing mtgenome information on Noctuoidea is very limited. To date, only 7 species have mtgenomes publicly available in GenBank. Erebidae was upgraded to family from Erebinae [9] within Noctuoidea and newly revised by Zahiri *et al*. [10]. Moreover, current genomic knowledge of which is even scantier which is limited to 3 species belonging to 2 subfamilies among 18 known. A better understanding of Noctuoidea or Erebidae all deeply requires an expansion of taxon and genome samplings using which to get datasets for a strong phylogenetic signal. Zahiri *et al*. (2011) proposed a newly robust phylogenetic framework of Noctuoidea with six families: Oenosandridae, Notodontidae, Erebidae, Euteliidae, Nolidae and Noctuidae, in which the relationship of Erebidae only a few lineages are well supported [11].

Ctenuchinina (Lepidoptera: Noctuoidea: Erebidae: Arctiinae) consists of four subtribes in two tribes: Syntomina and Thyretina in Syntomini as well as Euchromiina and Ctenuchina in Arctiini [9], [10], [11], which was formerly treated as an independent family named Ctenuchidae ( = Syntomidae, Euchromidae, Amatidae) (e.g. [12]). It is not a monophyletic group. Ctenuchinina contains a large number of diurnal moths which are phytophagous pests in agriculture and forest since the larvae and adults have massive economic impact on crop production and forest protection. *Cisseps fulvicollis*, for instance, has been recorded as an economic destructive insect on grain corn [13]. Hence, the resolution of a stable classificatory structure for the major lineages of these moths, and understanding their phylogenetic relationships, are meaningful to biological prevention and control.

Ctenuchinina was confused with the species of Zygaenidae and Sesiidae in the history, and fell into Sphingidae or Zygaenidae in early research. Herrich-Sch¨affer clearly separated this group from Zygaenidae and treated it as a family based on the type genus of *Syntomis* Ochsenheimer, 1808 which was the synonym of *Amata* Fabricius, 1807. The classification relationships of Ctenuchinina is based on the presences of a metepisternal tymbal organ, genitalic character, larvae and venation which failed to offer a clear conclusion since crossing synapomorphy is always inevitable existence. As the intricate relationship among itself as well as with close related groups, the classification status of Ctenuchinina presents long-term, constantly change. Aim to figure out some divergence in the morphological taxonomy, molecular characters were introducted to perform taxonomic studies of Ctenuchinina. But these studies are still very scant and were restricted to several molecular markers. Wink *et al*. used 16S rRNA sequences to construct phylogenetic relationships, in which Ctenuchidae was downgraded to subfamily status within Arctiidae [14]. Schneider *et al*. proposed a split of the genus *Amata* in two distinct genera based on mitochondrial 16S rRNA gene [15]. Therefore seeking more approach and genetic markers to slove these problems is become necessary effort.

In addition, the available gene knowledge of Ctenuchinina is limited and narrow as well exemplified by sequences available in GenBank that were obtained mostly cytochrome oxidase subunit 1 (COI) genes. There are more than 2800 sequences with about 2659(accounting for about 94.19%)are COI genes of very short length of 600–700 bp, and the remains are a handful of mRNA (about 6) and other sequences without any mtgenome. Undoubtedly, these nucleotide information is extremely limited relative to whether the entire mitochondrional length of 15–20 kb or the genes of 37 and a control region with variable length.

Considering the insufficient and perplexity above, in the present work, we sequenced, annotated and compared an entire mitogenome of *A. emma* (Lepidoptera: Erebidae) which would be the first complete mitochondrial genome of Ctenuchinina. What is more, we compared it with other lepidopteran genomes available so as to get conservation and variance information of Ctenuchinina relative to others, and infer a phylogenetic relationship of Lepidoptera with the expectation for providing robust molecular evidence for taxonomic status of Ctenuchinina, and providing robust information on understanding the phylogenetic relationships of Noctuoidea and Erebidae.

## Materials and Methods

### Sample collection and DNA extraction

One ethanol-preserved adult of *A. emma* was collected form an organic apple orchard in Beijing, China, in July 2011. Since this orchard is one of field stations for studying insect biodiversity, where there are no endangered or protected species and we have been working for about six years, no specific permits were required for our collecting. Total genomic DNA was extracted from the single sample with the DNeasy Blood &Tissue kit. The detailed procedures were consistent with the manufacturer instructions.

### PCR amplification, cloning and sequencing

**Figure 1:**
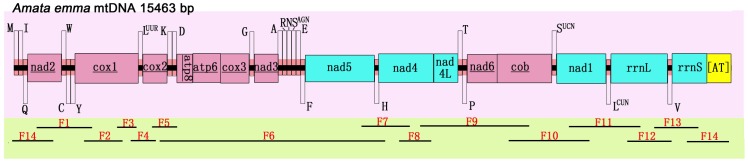
Map of the mitochondrial genome of *A. emma*. Protein-coding genes (names with underlines) coded on the majority strand are pink colored, while the rest and two rRNA genes coded on the minority strand are blue colored. The tRNA genes with single letter above the central axis are coded on majority strand. Underscores under the axis with F1–F14 indicate positions of 14 overlapping PCR amplified fragments.

In order to get the whole genome, 14 pairs of primers were used for PCR amplification. The full list of primers is showed in Table 1. Figure 1 provides the coverage areas of PCR fragments. Eight pairs of universal primers [16] were used to amplify fragments 4, 5, 6, 10, 11, 12, 13 and 14. Primer combination LCO1490 with HCO2198 was used to amplify fragment 2. Primers for fragment 3 were modified form Simon *et al.* [16]. As for the other fragments 1, 7, 8 and 9, primers were designed with Primer Premier 5.0 software. Sequences of *Phalera flavescens* (Accession: NC016067), *Sesamia inferens* (Accession: NC015835), *Helicoverpa armigera* (Accession: NC014668), *Hyphantria cunea* (Accession: NC014058), *Lymantria dispar* (Accession: NC012893), and *Ochrogaster lunifer* (Accession: NC011128) were downloaded from GenBank and aligned using Clustal X [17] to obtain the conserved sequence, which can provide references for designing PCR primers. All primers were synthesized by Shanghai Sangon Biotechnology Co., Ltd. (Beijing, China).

**Table 1:** Regions and primers in present paper.

Fragment (Region)		Primer (F/R)	Primer sequence (F/R) 5′→3′
F1	*nad2-cox1*	Nad2-J-416/cox1-N-1693	TTTACCCTCAACTGAAGCCTCCT/TACTAATCAGTTACCAAATCCTCCA
F2	*cox1*	Lco1490/Hco2198	GGTCAACAAATCATAAAGATATTGG/TAAACTTCAGGGTGACCAAAAAATCA
F3	*cox1-trnL2*	C1-J-1751/TL2-N-3014	GGATCACCTGATATAGCATTCCC/TCCAATGCACTAATCTGCCATATTA
F4	*cox1-cox2*	C1-J-2797/C2-N-3494	CCTCGACGTTATTCAGATTACC/GGTAAAACTACTCGATTATCAAC
F5	*cox2-trnD*	C2-J-3400/A8-N-3914	ATTGGACATCAATGATATTGA/TCATCTTATAGGTACTATTTGAGG
F6	*cox2-nad4*	C2-J-3696/N4-N-8484	GAAATTTGTGGAGCAAATCATAG/GCTAATATAGCAGCTCCTCC
F7	*nad5-nad4*	Nad5-J-7745/nad4-N-8820	TAAACCTAACCCATCTCACCCC/GGTTATGGGCTTTTACGATT
F8	*nad4*	Nad4-J-8569/nad4-N-9105	GCTAAACAAAATATCCCCGATGAAC/GTATCAGCCTGAGCGAATTAAAGCA
F9	*nad4-cob*	Nad4-J-8887/cob-N-11326	GGAGCTTCAACATGAGCTTT/GCATAAGCAAATAAGAAATATCATTC
F10	*cob-nad1*	CB-J-10933/N1-N-12595	TATGTACTACCATGAGGACAAATATC/GTAGCATTTTTAACTTTATTAGAACG
F11	*nad1-rrnL*	N1-J-12585/LR-N-13398	GGTCCCTTACGAATTTGAATATATCCT/CGCCTGTTTAACAAAAACAT
F12	*rrnL-rrnS*	LR-J-12887/SR-N-14588	CCGGTCTGAACTCAGATCACGT/AAACTAGGATTAGATACCCTATTAT
F13	*rrnL-rrnS*	LR-J-13331/SR-N-14756	TGATTATGCTACCTTTGCACAGT/GACAAAATTCGTGCCAGCAGT
F14	*rrnS-nad2*	SR-J-14612/N2-N-732	AGGGTATCTAATCCTAGTTT/GAAGTTTGGTTTAAACCTCC

PCR amplification conditions were as follows: an initial denaturation for 5 min at 95°C, followed by 35 cycles of denaturation for 30 s at 94°C, annealing for 30 s at 48–55°C (depending on primer combination), elongation for 1–3 min (depending on putative length of the fragments) at 68°C, and a final extension step of 72°C for 10 min. All amplifications applied Takara LA Taq (Takara Co., Dalian, China) and performed on an Eppendorf Mastercycler and Mastercycler gradient.

The PCR products were resolved by electrophoresis in 1.0% agarose gel, purified using 3Spin PCR Product Purification Kit. All amplified products except *rrnS-nad2* were sequenced directly using upstream and downstream primers along both strands by ABI-377 automatic DNA sequencers. The *rrnS-nad2* fragment was sequenced after being ligated to the p*EASY*-T3 Cloning Vector (Beijing TransGen Biotech Co., Ltd., Beijing, China), and then sequenced by M13-F and M13-R primers and walking. Sequencing was performed using ABI BigDyever 3.1 dye terminator sequencing technology and run on ABI 3730XL PRISM 3730 × 1capillary sequencers. All sequencing procedures repeated at least three times.

### Sequence assembling and annotation

The overlapping PCR product sequences were checked and assembled using BioEdit [18] and DNAStar package DNAStar package (DNAStar Inc. Madison, USA). Rough locations of genes were initially identified via BLAST on NCBI and comparison with the other lepidopteran sequences available in GenBank.

The protein-coding sequences were translated into putative proteins on the basis of the Invertebrate Mitochondrial Genetic Code. Composition skew analysis was carried out according to formulas AT skew = [A-T]/[A+T] and GC skew = [G-C]/[G+C], respectively [19]. The A+T content and Relative Synonymous Codon Usage (RSCU) were calculated by MEGA [20].

The tRNA genes were indentified using the tRNAscan-SE Search [21] or predicted by sequence features of being capable of folding into the typical cloverleaf secondary structure with legitimate anticodon, and their secondary structures were drawn by RNAstructure program [22].

The secondary structure of *rrnS* and *rrnL* were inferred from models proposed for other insects. XRNA 1.2.0.b (http://rna.ucsc.edu/rnacenter/xrna/xrna.html) was used to draw the folding structure with the reference of the results of the CRW site [23] and other insect species. The tandem repeats of A+T-rich region were found via the Tandem Repeats Finder program, and the stem-loop structure was determined by the Mfold Web Server [24].

### Phylogenetic analysis

To construct a phylogenetic relationship of Lepidoptera, 54 complete or near-complete lepidopteran mitogenomes were downloaded from GenBank (Table 2). Besides, mitogenomes of *Bactrocera oleae* (NC_005333) [25] and *Anopheles gambiae* (NC_002084) [26] were downloaded and used as outgroups of the 55 taxa including the one we sequenced presently.

**Table 2:** List of taxa analyzed in present paper.

Subfamily	Family	Species	Length	Acc.number	Reference
Bombycoidea	Bombycidae	*Bombyx mori*	15,643 bp	NC_002355	Lee et al., unpulished
		*Bombyx mandarina*	15,928 bp	NC_003395	[46]
	Saturniidae	*Antheraea pernyi*	15,566 bp	NC_004622	[47]
		*Antheraea yamamai*	15,338 bp	NC_012739	[48]
		*Samia cynthia ricini*	15,384 bp	NC_017869	[49]
		*Saturnia boisduvalii*	15,360 bp	NC_010613	[50]
		*Eriogyna pyretorum*	15,327 bp	NC_012727	[51]
		*Actias selene*	15,236 bp	NC_018133	[52]
	Sphingidae	*Manduca sexta*	15,516 bp	NC_010266	[31]
Geometroidea	Geometridae	*Phthonandria atrilineata*	15,499 bp	NC_010522	[53]
Noctuoidea	Noctodontidae	*Phalera flavescens*	15,659 bp	NC_016067	[54]
		*Ochrogaster lunifer*	15,593 bp	NC_011128	[3]
	Erebidae	*Lymantria dispar*	15,569 bp	NC_012893	[55]
		*Hyphantria cunea*	15,481 bp	NC_014058	[42]
		***Amata emma***	15,463 bp	KC_513737	The present study
	Noctuidae	*Helicoverpa armigera*	15,347 bp	NC_014668	[43]
		*Sesamia inferens*	15,413 bp	NC_015835	Chai et al., unpublished
Pyraloidea	Crambidae	*Ostrinia nubilalis*	14,535 bp	NC_003367	[56]
		*Diatraea saccharalis*	15,490 bp	NC_013274	[57]
		*Ostrinia furnacalis*	14,536 bp	NC_003368	[56]
		*Chilo suppressalis*	15,395 bp	NC_015612	[35]
		*Cnaphalocrocis medinalis*	15,388 bp	NC_015985	[35]
	Pyralidae	*Corcyra cephalonica*	15,273 bp	NC_016866	Wu et al., unpublished
Tortricoidea	Tortricidae	*Adoxophyes honmai*	15,680 bp	NC_008141	[39]
		*Grapholita molesta*	15,717 bp	NC_014806	[58]
		*Spilonota lechriaspis*	15,368 bp	NC_014294	[41]
Papilionoidea	Papilonidae	*Papilio machaon*	15,185 bp	NC_018047	Xu et al., unpublished
		*Papilio bianor*	15,340 bp	NC_018040	Xu et al., unpublished
		*Teinopalpus aureus*	15,242 bp	NC_014398	[59]
		*Parnassius bremeri*	15,389 bp	NC_014053	[60]
		*Papilio maraho*	16,094 bp	NC_014055	Wu et al., unpublished
	Nymphalidae	*Euploea mulciber*	15,166 bp	NC_016720	[61]
		*Libythea celtis*	15,164 bp	NC_016724	[61]
		*Melitaea cinxia*	15,170 bp	NC_018029	Xu et al., unpublished
		*Issoria lathonia*	15,172 bp	NC_018030	Xu et al., unpublished
		*Kallima inachus*	15,183 bp	NC_016196	[62]
		*Acraea issoria*	15,245 bp	NC_013604	[63]
		*Argynnis hyperbius*	15,156 bp	NC_015988	[64]
		*Apatura ilia*	15,242 bp	NC_016062	[65]
		*Sasakia charonda*	15,244 bp	NC_014224	Hakozaki et al., unpublished
		*Hipparchia autonoe*	15,489 bp	NC_014587	[66]
		*Apatura metis*	15,236 bp	NC_015537	[67]
		*Sasakia charonda kuriyamaensis*	15,222 bp	NC_014223	Hakozaki et al., unpublished
		*Athyma sulpitia*	15,268 bp	NC_017744	[68]
		*Calinaga davidis*	15,267 bp	NC_015480	[69]
		*Fabriciana nerippe*	15,140 bp	NC_016419	[70]
	Pieridae	*Pieris rapae*	15,157 bp	NC_015895	[71]
		*Pieris melete*	15,140 bp	NC_010568	[72]
		*Aporia crataegi*	15,140 bp	NC_018346	[73]
	Lycaenidae	*Coreana raphaelis*	15,314 bp	NC_007976	[36]
		*Spindasis takanonis*	15,349 bp	NC_016018	[74]
		*Protantigius superans*	15,248 bp	NC_016016	[74]
Yponomeutoidea	Lyonetiidae	*Leucoptera malifoliel*	15,646 bp	JN_790955	[45]
Hepialoidea	Hepialidae	*Thitarodes renzhiensis*	16,173 bp	NC_018094	[32]
		*Ahamus yunnanensis*	15,816 bp	NC_018095	[32]

Two analytical approaches, Maximum Likelihood (ML) and Bayesian Inference (BI), were used to infer phylogenetic trees. Nucleotide sequences of each of the 13 PCGs were translated into amino acid sequences then aligned with default settings by MEGA, and these 13 resultant alignments were retranslated into nucleotide alignments by MEGA separately. These processed alignments were concatenated together by BioEdit and thus got a nucleotide matrix of 11,751 sites in length. Substitution model selection was conducted by MrModeltest2.3 (http://www.abc.se/~nylander/mrmodeltest2/mrmodeltest2.html) [27]. The Bayesian analyse was performed with MrBayes [28] for Bayesian while ML analysis was performed by RAxML [29] for likelihood, and GTR + I +G model was the appropriate model of molecular evolution. The Bayesian analyse under the following conditions: 1,000,000 generations, 4 chains (1 cold chain and 3 hot chains) and a burn-in step for the first 10,000 generations. The confidence values of the BI tree were expressed as the Bayesian posterior probabilities in percentages. The ML analysis was performed using default parameters and the confidence values of the ML tree were evaluated via a bootstrap test with 1000 iteration.

## Results and Discussion

### Genome structure and organization

The *A. emma* (GenBank accession : KC_513737) mitogenome is a closed-circular molecule of 15,463 bp. It contains the typical set of 37 genes (13 PCGs, 22 tRNAs and 2 rRNAs) as in most animal mtDNA [1]. Gene order and orientation of *A. emma* are identical to the other ditrysian insects to date, and the locations of *trnM* gene follow the ditrysian type *trnM-trnI-trnQ* [30], [30,31] which is different from non-ditrysian groups in Lepidoptera [32]. Twenty-three genes are coded on the majority strand while the rest are coded on the minority strand (Table 3 and Figure 1).

**Table 3:** Summary of mitogenome of *Amata emma*.

Gene	Direction	Form	To	Size	Inc	Anticodon	Start codon	Stop codon
*trnM*	F	1	68	68	6	CAT	——	——
*trnI*	F	75	140	66	0	GAT	——	——
*trnQ*	R	141	209	69	51	TTG	——	——
*nad2*	F	261	1274	1014	1	——	ATT	TAA
*trnW*	F	1276	1343	68	−8	TCA	——	——
*trnC*	R	1336	1398	63	6	GCA	——	——
*trnY*	R	1405	1470	66	7	GAT		
*cox1*	F	1478	3011	1534	0	——	CGA	T-*trnL2*
*trnL2*(UUR)	F	3012	3079	68	0	TAA	——	——
*cox2*	F	3080	3759	680	0	——	ATG	TA-*trnK*
*trnK*	F	3760	3830	71	−1	CTT	——	——
*trnD*	F	3830	3909	78	−10	GTC	——	——
*atp8*	F	3900	4076	177	−7	——	ATT	TAA
*atp6*	F	4070	4747	678	5	——	ATG	TAA
*cox3*	F	4753	5541	789	2	——	ATG	TAA
*trnG*	F	5544	5609	66	0	TCC	——	——
*nad3*	F	5610	5963	354	3	——	ATT	TAA
*trnA*	F	5967	6032	66	−1	TGC	——	——
*trnR*	F	6032	6094	63	0	TCG	——	——
*trnN*	F	6095	6160	66	4	GTT	——	——
*trnS1*(AGN)	F	6165	6230	66	0	TCT	——	——
*trnE*	F	6231	6297	67	10	TTC	——	——
*trnF*	R	6308	6373	66	0	GAA	——	——
*nad5*	R	6374	8116	1743	0	——	ATA	TAA
*trnH*	R	8117	8182	66	0	GTG	——	——
*nad4*	R	8183	9521	1339	0	——	ATG	T-*nad4L*
*nad4L*	R	9522	9809	288	5	——	ATG	TAA
*trnT*	F	9815	9880	66	0	TGT	——	——
*trnP*	R	9881	9946	66	8	TGG	——	——
*nad6*	F	9955	10488	534	9	——	ATA	TAA
*cob*	F	10498	11652	1155	6	——	ATG	TAA
*trnS2*(UCN)	F	11659	11725	67	20	TAG	——	——
*nad1*	R	11746	12684	939	1	——	ATG	TAA
*trnL1*(CUN)	R	12686	12753	68	0	TAC	——	——
*rrnL*	R	12754	14124	1371	0	——	——	——
*trnV*	R	14125	14189	65	0	——	——	——
*rrnS*	R	14190	14981	792	0	——	——	——
A+T-rich region		14982	15463	482	0	——	——	——

*Inc* = intergenic nucleotides.

The genome composition (A: 37.8%, T: 40.8%, C: 13% and 7.5%) of the major strand shows highly A+T biased which accounts for 79.5%, and exhibits negative AT-skew (−0.026) and GC-skew (−0.268). As for the other lepidopteran mitochondrion genomes previously sequenced, the value of AT-skew (−0.026) is in the range from −0.06 (*Bombyx mori*) to 0.05 (*Athyma sulpitia*) while the GC-skew (−0.268) is in the range from −0.32 (*Ochrogaster lunifer*) to −0.16 (*C. raphaelis*). The full list of composition and skewness of *A. emma* is shown in Table 4.

**Table 4:** Composition and skewness of *A. emma* mitochondrional genome regions.

nt %	Whole mtDNA	Protein-coding sequence			rRNAs	tRNAs	IGs		
		1^st^ #	2^nd^ #	3^rd^ #			IGs	A+T-rich	Short-IGs
A%	38.7	36.8	22.0	41.1	38.9	40.4	42.3	42.9	40.3
T%	40.8	36.3	48.3	48.8	44.8	40.2	49.7	49.8	49.3
C%	13.0	10.5	16.4	6.1	11.5	11.5	5.3	4.4	8.3
G%	7.5	16.4	13.2	4.0	4.7	7.9	2.7	2.9	2.1
A+T%	79.5	73.1	70.3	89.9	83.7	80.6	92	92.7	89.6
C+G%	20.5	26.9	29.6	10.1	16.2	19.4	8.0	7.3	10.4
AT-Skew%	−0.026	0.007	−0.374	−0.086	−0.07	0.002	−0.08	−0.074	−0.1
GC-skew%	−0.268	0.219	−0.108	−0.207	−0.42	−0.186	−0.325	−0.205	−0.596

# = position.

*IGs* = non-coding intergenic spacer regions.

### Protein-coding genes

**Figure 2:**
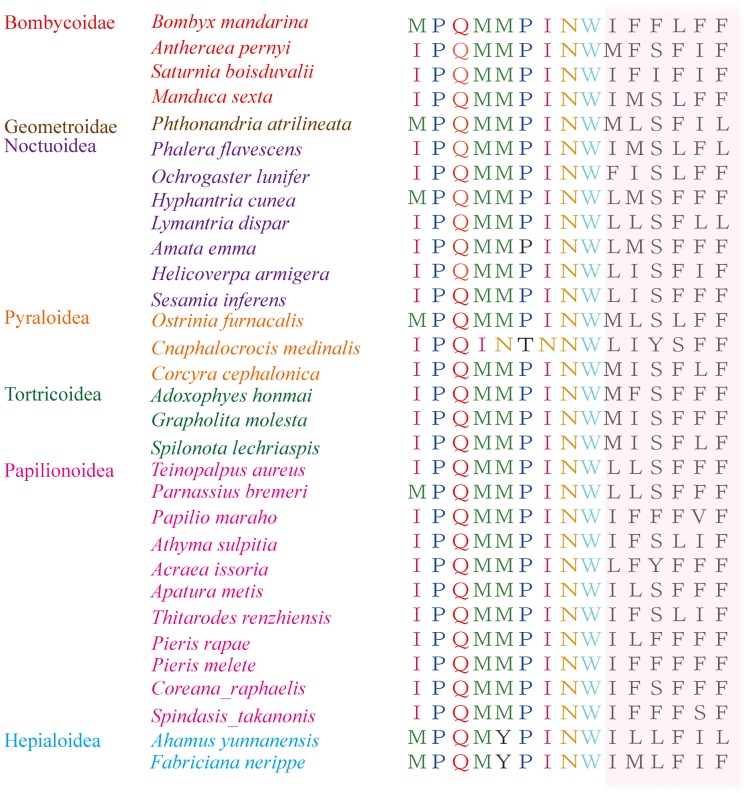
The highly conserved sequence of 5′ end of atp8 gene among seven superfamilies in Lepidoptera.

Among 13 protein-coding genes, nine (*nad2*, *cox1*, *cox2*, *atp8*, *atp6*, *cox3*, *nad3*, *nad6* and *cob*) are coded on the majority strand while the rest (*nad5*, *nad4*, *nad4*L, *nad1*) are coded on the minority strand. The initial codons are the canonical putative start codons ATN (ATA for *nad5*, *nad6*; ATT for *nad2*, *atp8*, *nad3*; ATG for *cox2*, *atp6*, *cox3*, *nad4*, *nad4L*, *cob*, *nad1*), with the exception of *cox1*gene which uses CGA instead. A recent study has used expressed sequence tag to explain that *cox1* may start with CGA [33]. Though controversy exists for the start codon of *cox1*, the present study shows the use of CGA. Ten genes share complete termination codon TAA, and three genes use incomplete stop codons (a single T for *cox1* and *nad4*, TA for *cox2*). The non-canonical stop codons will be corrected via post-transcriptional polyadenylation [34]. The *atp8* and the *atp6* have a 7 bp overlap, which is common to all Lepidoptera mitogenomes known to date [3], [32]. The 5′ end of *atp8* gene is highly conserved in Lepidoptera-IPQMMINW or MPQMMINW, and *A. emma* also presents this characteristic with no exception (Figure 2).

The A+T content of three codon positions of the PCGs was calculated (the stop codons were excluded from the analysis) and is showed in Table 4. The third position has a relatively high A +T content (89.9%), while the first and the second positions have 73.1% and 70.3%, respectively. In addition, both the second and the third position have negative AT-skew and GC-skew.

**Figure 3:**
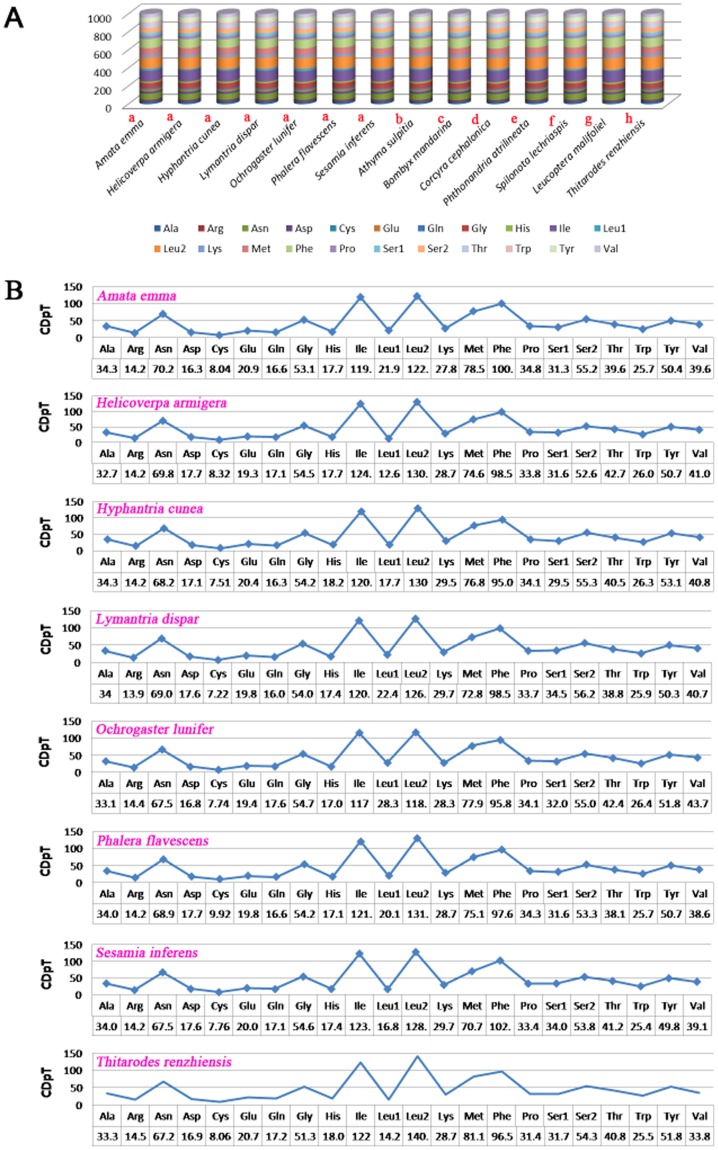
Codon distribution. A: Comparison of the codon usage of mitochondrial genome across eight superfamilies in Lepidoptera. The lowercase alphabet (a, b, c, d, e, f, g and h) above the species name represent the superfamily the species belong to (a:Noctuoidea, b: Papilionoidea, c: Bombycoidea, d: Pyraloidea, e: Geometroidea, f: Tortricoidea, g: Yponomeutoidea, h: Hepialoidea). B: Codon distribution in Noctuoidae. CDspT, codons per thousand codons.

Comparison results of the codon usage of mitochondrial genomes across eight superfamilies of Lepidoptera are showed in Figure 3A. Fourteen species in Lepidoptera (seven belonging to Noctuoidae, the rest belonging to Bombycoidae, Geometroidae, Pyraloidea, Tortricoidea, Papilionoidea, Yponomeutoidea and Hepialoidea, respectively) (Figure 3A) were examined and the results show that *Leu2*, *Ile*, *Phe*, *Met*, and *Asn* are the five most frequent amino acids. *Leu2*, as a hydrophobic amino acid, has the highest usage rate, which may relate to the function of chondriosome of encoding many transmembrane proteins. The rarest used codon family is *Cys*. Codon distributions of seven species in Noctuoidae are consistency and each amino acid has equal content in different species (Figure 3B).

**Figure 4:**
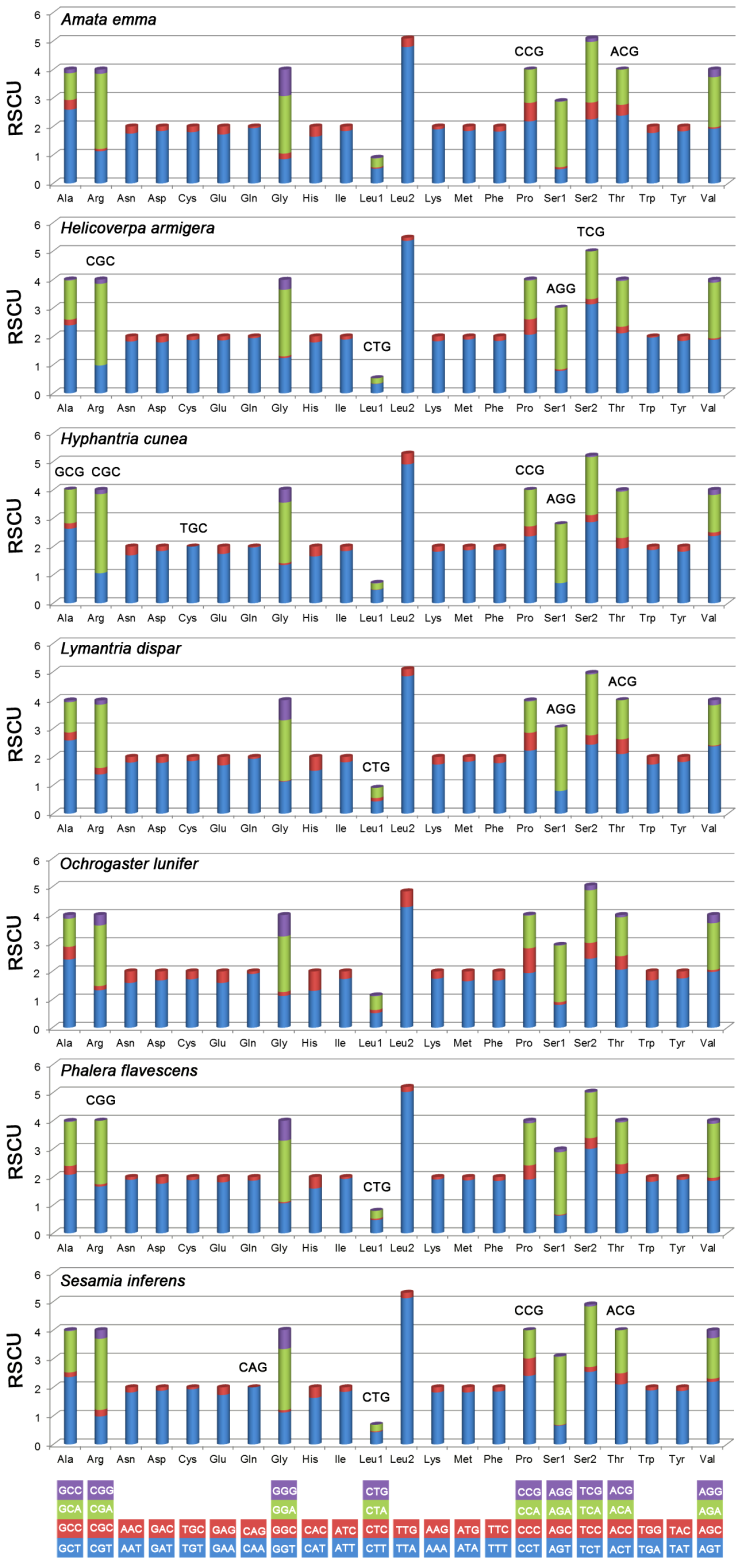
Relative Synonymous Codon Usage (RSCU) in Noctuoidae. Codon families are provided on the X axis. The codon above the bar indicate the one is not present in the genome.

RSCU for Noctuoidae is present in Figure 4. The usage of both two-fold and four-fold degenerate codon is biased to use the codons which are abundant in A or T in third position. The codons which have relatively high content of G and C are likely to be abandoned, which is consistent with other lepidopteran insects [35].

### Transfer RNA and ribosomal RNA genes

**Figure 5:**
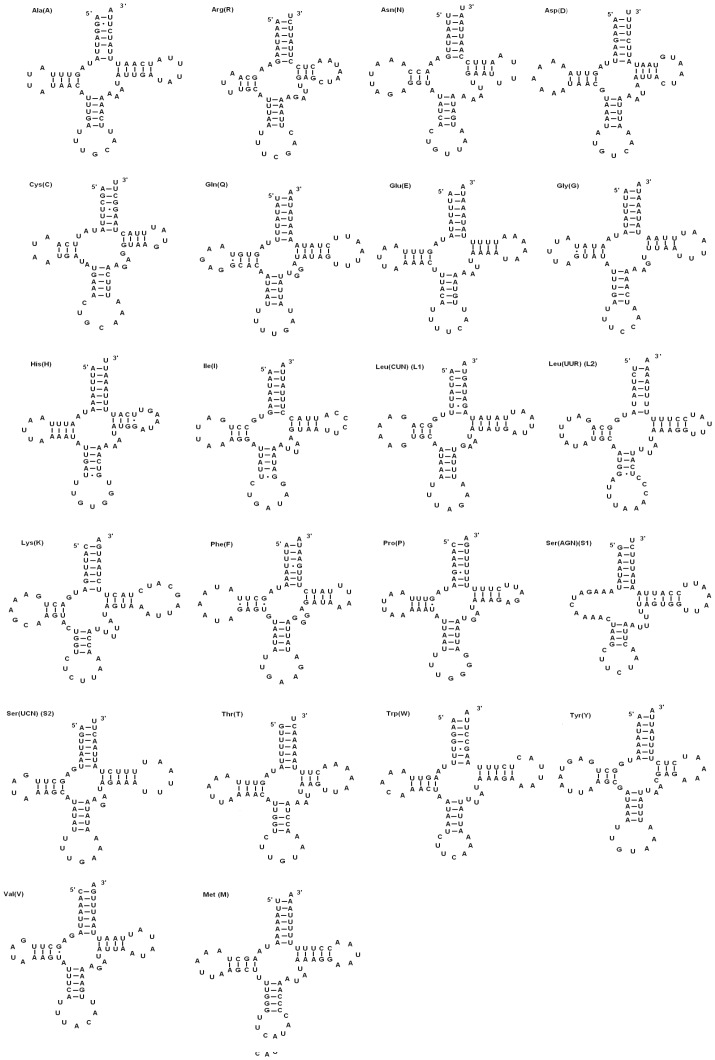
Predicted secondary structures for 22 tRNA genes of *A.emma* mitogenome. The tRNAs are labeled with the abbreviations of their corresponding amino acids. Dashes (−) indicate Watson-Crick base pairing and centered dots (·) indicate G-C base pairing.

The *A. emma* mitogenome contains the set of 22 tRNAs genes (Figure 5) as most of lepidoptera mtDNAs though the feature is not very Conserved in the animal mtDNAs, for examples, the lepidopteran insect *Coreana rapaelia* (NC_013604, [36]) have an extra *trnS1* (AGN) and another remarkable exceptions is the entire genus *Chrysomya* possessed duplicate *trnI* gene [37] such as *Chrysomya chloropyga* (NC_002697, [38]) have an extra *trnS1* (AGN) and *trnI*. The tRNAs are scattered throughout the circular molecule and vary from 63 bp (*trnC* and *trnR*) to 78 bp (*trnD*) in size, and show highly A+T biased, accounting for 80.6% and exhibit positive AT-skew (0.002). Among these tRNA genes, fourteen tRNAs are coded by the H-strand with the rest by the L-strand.

All tRNA genes have typical cloverleaf secondary structures, except for the *trnS1* (AGN) gene, in which the dihydrouridine (DHU) arm is simplified down to a loop. These features are common in most animal mitogenome, but exception does exist: *Adoxophyes honmai* tRNAs show complete clover leaf secondary structures [39].

The anticodons of *A. emma* tRNAs are all identical to most Lepidopteran mitogenomes, except for *trnS1*(AGN) which uses TCT instead of GCT as *Coreana rapaelia* [39], *Thitarodes renzhiensis* and *T. yunnanensis* [32].

A total of 24 mismatched base pairs and G-U wobble pairs scatter throughout the 16 tRNA genes (the amino acid acceptor (11), DHU (6), TψC (3), and anticodon stems (4)). The types are as follows: 8 mismatched base pairs (3 A–C and 5 U-U) and 16 G-U wobble pairs. The mismatched base pairs are corrected via RNA-editing mechanisms [40].

**Figure 6:**
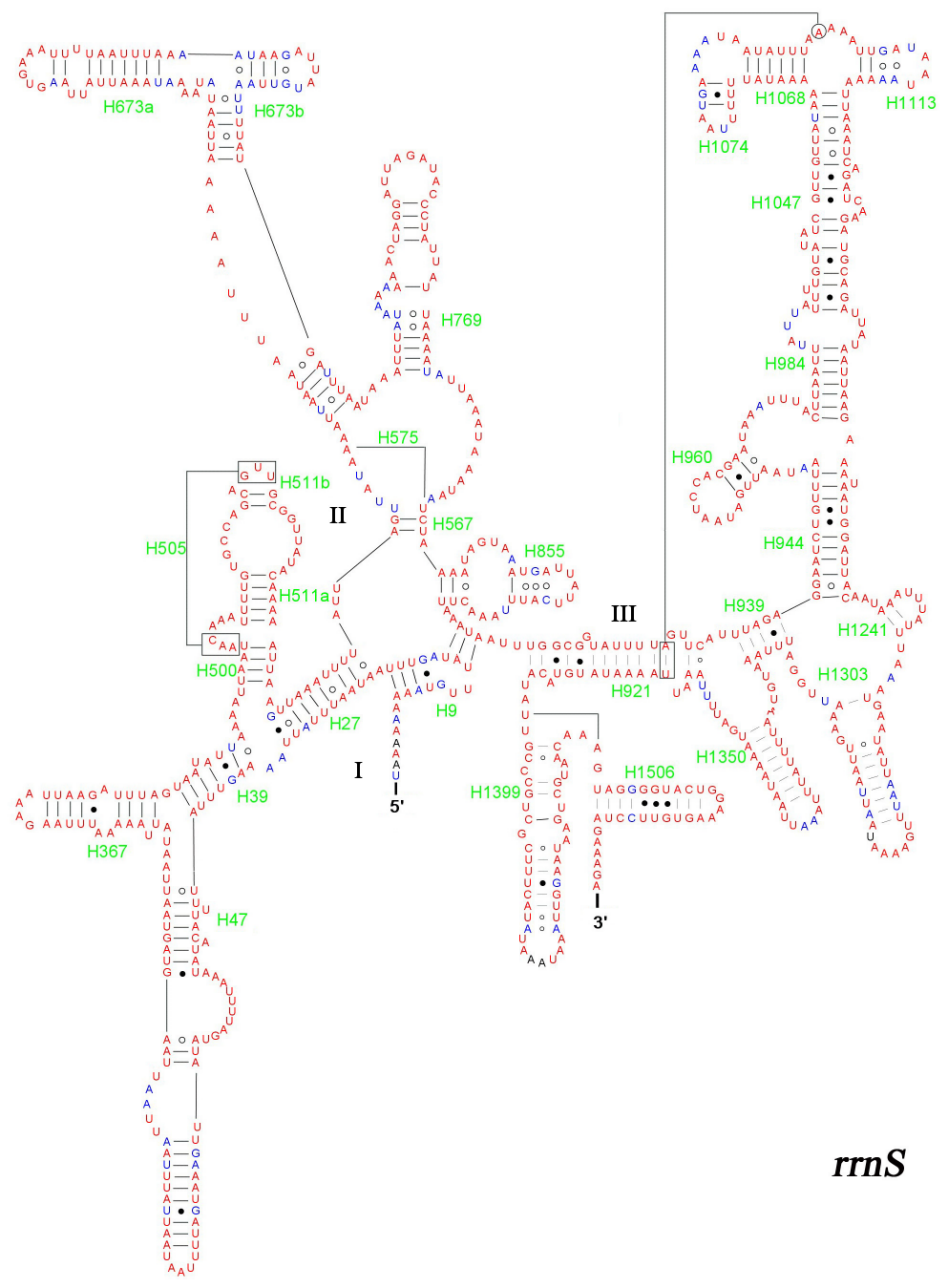
Predicted *rrnS* secondary structure in *A. emma* mitogenome. Tertiary interactions and base triple are shown connected by continuous lines. Dashes indicate Watson-Crick base pairing, centered dots indicate G-C base pairing and circles indicate other non-canonical pairs.

**Figure 7:**
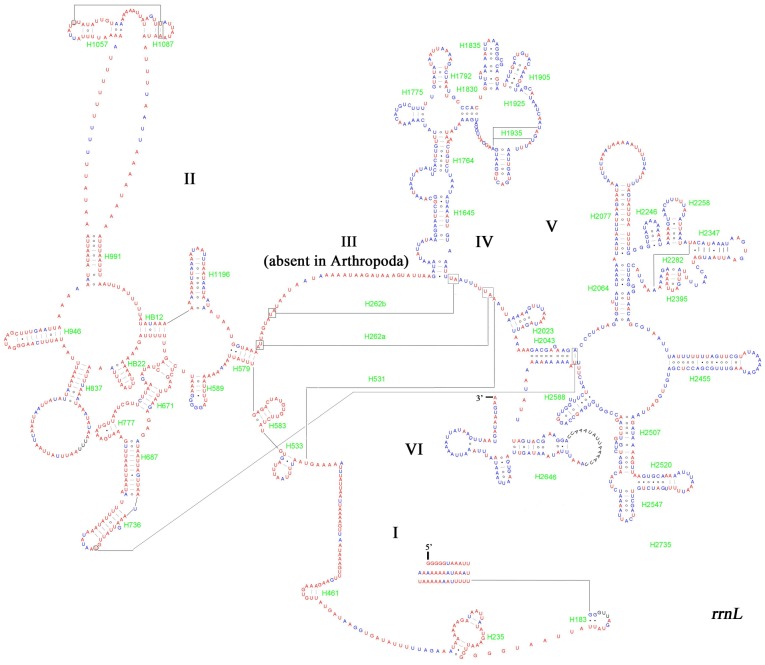
Predicted *rrnL* secondary structure in *A. emma* mitogenome. Tertiary interactions and base triple are shown connected by continuous lines. Dashes indicate Watson-Crick base pairing, centered dots indicate G-C base pairing and circles indicate other non-canonical pairs.

The two ribosomal RNA genes with 83.7% A+T content in total (Table 4) are located between *trnL1* and *trnV*, *trnV* and the A+T-rich region, respectively. The *rrnL* is 1371 bp while *rrnS* is 792 bp. The *rrnL* (Figure 6) has six domains (domain III is absent) and *rrnS* (Figure 7) has three. Both the secondary structures of two rRNA genes broadly conform with the secondary structure models proposed for these genes from other insects.

### Non-coding and overlapping genes

The non-coding regions of mtDNA of *A. emma* is 144 bp in total, is highly A+T biased (92.0%) (Table 3 and 4), and made up of 16 intergenic spacer sequences, ranging from 1 bp to 51 bp. There are three major intergenic spacers at least 10 bp in length (S1, S2 and S3). The S1 spacer (51 bp), located between *trnQ* and *nad2*, is common in lepidopteran mtDNA. The S2 spacer (10 bp), between *trnE* and *trnF*, varies widely in Lepidopteran insects. For instance, *trnE* and *trnF* have a 7 bp overlap in *Lechriaspis meyrick* [41], while in the mtDNA of *Ochrogaster lunifer* [3], the length of the spacer was 70 bp. The S3 spacer (20 bp), located between the *trnS2* and *nad1*, contains the “ATACTAA” motif, which is a common feature across Lepidopteran insects [31], [42]. This special motif was proposed to be a recognition site performed by mtTERM protein [2].

**Figure 8:**
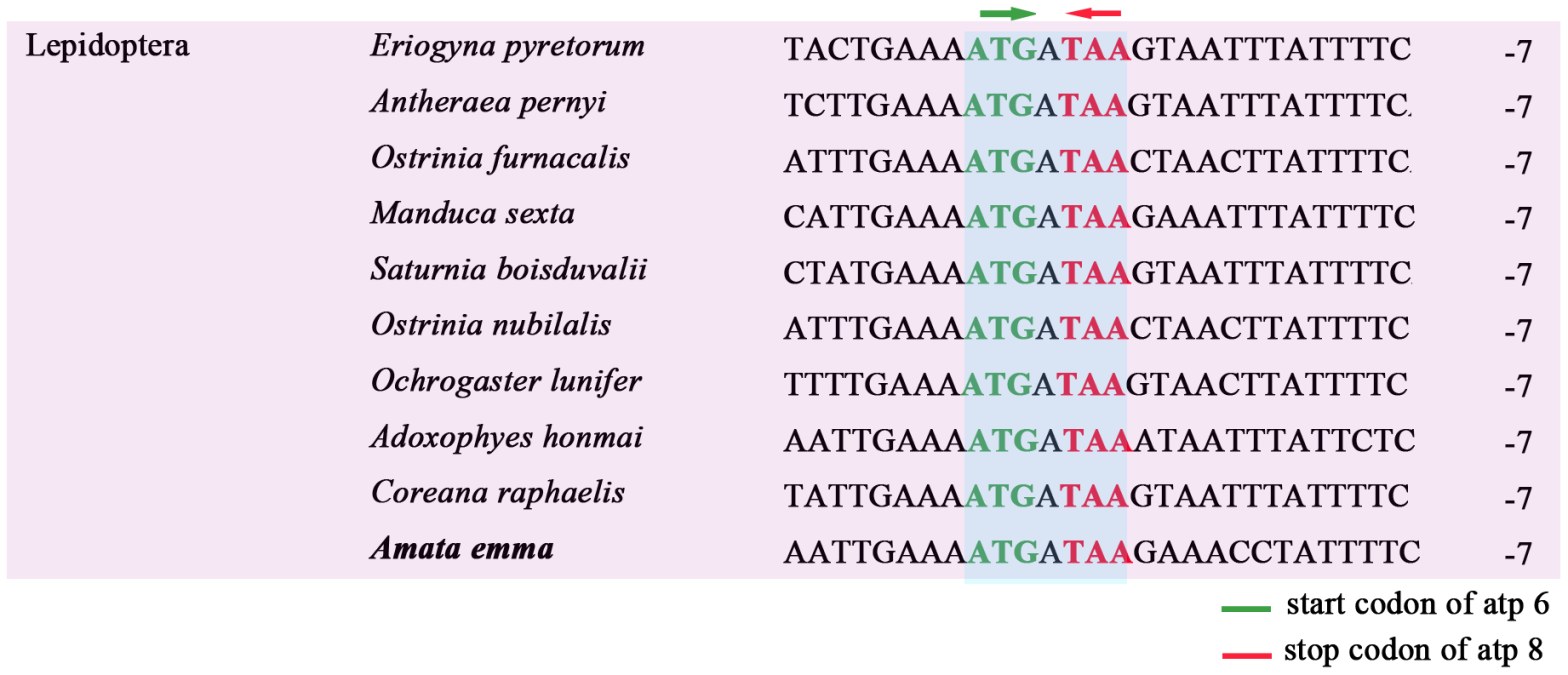
Alignment of overlapping region between *atp8* and *atp6* across Lepidoptera and other insects. The numbers on the right refer to intergenic nucleotides.

In addition, there are 4 overlapping regions belonging to two types of locations: between tRNA and tRNA (*trnW* and *trnC*, *trnK* and *trnD*, *trnA* and *trnR*) and protein and protein (*atp6* and *atp8*). The *atp8* and *atp6* have a 7 bp overlap, which is common in Lepidoptera mitogenomes. The intergenic nucleotides between *atp8* and *atp6* belonging to 10 species of Lepidoptera were examined and shown in Figure 8. Strikingly, these seven nucleotides “ATGATAA” is a commom feature across lepidoptera mtgenome.

### The A+T-rich region

The A+T-rich region, located between *rrnS* and *trnM*, spans 482 bp. The region contains 92.7% AT nucleotides,with negative AT skew and GC skew. The pattern of a motif “ATAGA” following *rrnS* and followed by 18–22 bp poly-T stretch which is considered to be a gene regulation element is a common feature occurring in Lepidoptera [3], [41] and in *A. emma*, the motif “ATAGA” located 17 bp downstream from *rrnS* and the poly-T stretch is 19 bp in length. A poly-A (in majority strand) is present upstream *trnM* in most Lepidopteran insects, but *A. emma* does not have the motif, and shares the feature with another lepidopteran insect *Helicoverpa armigera* [43]. In addition, the region of *A. emma* lacks conspicuous long repeated segments and just has several short repeats. The potential stable stem-and-loop structures were detected in AT region, which are inferred to be gene regulation elements. A microsatellite preceded by the ‘ATTTA’ motif is common across the region of Lepidopteran mitogenomes (e.g. *Ochrogaster lunifer*, [3]). In *A. emma*, (AT)_9_ element preceded by the ‘ATTTA’ motif is present in the 3′ end of the *A. emma* A+T- rich region. (AT)_n_GTAT is another feature of *A. emma* and there are three DNA fragments able to form this type of structures [(AT)_9_GTAT, (AT)_7_GTAT and (AT)_10_GTAT]. These structures could be the result of miss-pairing duplication [3].

### Phylogenetic relationships

**Figure 9:**
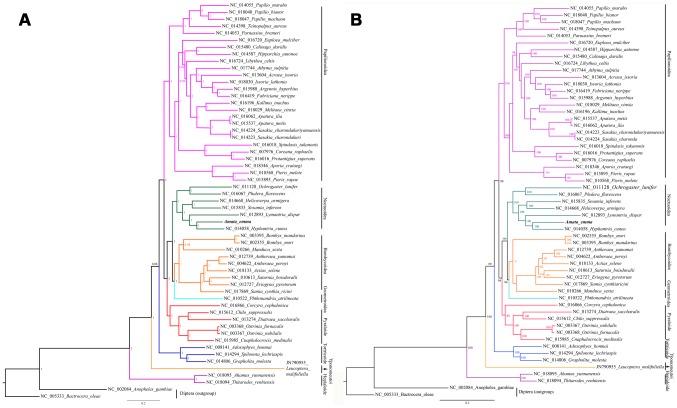
Inferred phylogenetic relationship among Lepidoptera based on amino acid sequence of mitochondrional 13 PCGs using Bayesian Inference (BI) (A) and maximum likelihood (ML) (B). Number at each node show posterior probabilities (A) and bootstrap percentages (B), respectively. *Bactrocera oleae* (NC_005333) and *Anopheles gambiae* (NC_002084) were used as outgroups.

Our analyses are based on sequence data from 13 protein-cording gene regions derived from 55 lepidopteran insects. Data matrix (11,751 bp of total) was analyzed by model-based evolutionary methods (Bayesian Inference and Maximum Likelihood) (Figure 9A and 9B).

The optimal cladograms infered by these two methods are very similar which are agree almost perfectly with he previously obtained by other studies [44], [45], however the nodes have a higher support and thus many interrelationships are well-resolved within Lepidoptera.

It is clearly that *A. emma* shares a close ancestry with *Hyphantria cunea* with quite well supported both by BI and ML analysis. Our findings provide strong support ( = 100;  = 1) for the monophyly of Noctuoidea which is higher than Zahiri et al [12]. Some traditionally families and subfamilies show clear evolutionary relationship with strong posterior probabilities and bootstrap support. For example, there is well-support for a clade with Notodontidae as sister to another well-support clade comprising Noctuidae + Erebidae. Erebidae comes out as a well-supported (posterior probabilities = 1; bootstrap = 100) monophyletic clade, which Lymantriinae (represented by *Lymantria dispar*) and Arctiinae (represented by *Hyphantria cunea* and *Amata emma*) are clearly confirmed as belonging to.

Within Papilionoidea, the clade comprising Pieridae and (Lycaenidae + Nymphalidae) form a separate but lower-supported lineage in ML method while well support (bootstrap<50) by BI method (posterior probabilities = 1). To confirm these relationships, more studies need to be performed.

In addition, there is rather strong support (posterior probabilities >0.9; bootstrap>80) for the clade of Bombycoidea, Pyralioidea, Tortricoidea and Hepialidae. However, for Geometroidea, though the support is well, the result really requires advanced studies based on massive samples to provide a robust phylogenetic framework.

## Conclusion

In this study, the mtgenome of *Amata emme* was sequenced, analyzed and compared with other lepidopteran insects, which would be the first whole mtgenome record of Ctenuchinina. The mtgenome shares many features with those of most Lepidopteran instects reported previously, just with some subtle differences in A+T region. In addition, we clarified the taxonomic status of Ctenuchinina using model-based phylogenetic inference and thus provide evidence for biological protection based on molecular markers.

The phylogenetic relationships based on nucleotide sequences of 13 PCGs using Bayesian inference and maximum likelihood methods provided a well-supported a broader outline of Lepidoptera and which agree with the traditional morphological classification and recently working, but with a much higher support. In this study, despite we have not performed much process on data matrix such as partition by codes, the result really provide a robust phylogenetic framework, which may imply that 13PCGs which have the function of express protein determining biological trait can be used as materials for phylogenetic inference just under a simple organization. However, this implication deeply needs more studies to verify whether it is universally applicable or not.
